# FOCUS mHealth Intervention for Veterans With Serious Mental Illness in an Outpatient Department of Veterans Affairs Setting: Feasibility, Acceptability, and Usability Study

**DOI:** 10.2196/26049

**Published:** 2022-01-28

**Authors:** Benjamin Buck, Janelle Nguyen, Shelan Porter, Dror Ben-Zeev, Greg M Reger

**Affiliations:** 1 Behavioral Research in Technology and Engineering (BRiTE) Center Department of Psychiatry and Behavioral Sciences University of Washington Seattle, WA United States; 2 VA Puget Sound Healthcare System Seattle, WA United States

**Keywords:** mHealth, veterans, schizophrenia, serious mental illness, mobile phone

## Abstract

**Background:**

Veterans with serious mental illnesses (SMIs) face barriers to accessing in-person evidence-based interventions that improve illness management. Mobile health (mHealth) has been demonstrated to be feasible, acceptable, effective, and engaging among individuals with SMIs in community mental health settings. mHealth for SMIs has not been tested within the Department of Veterans Affairs (VA).

**Objective:**

This study examines the feasibility, acceptability, and preliminary effectiveness of an mHealth intervention for SMI in the context of VA outpatient care.

**Methods:**

A total of 17 veterans with SMIs were enrolled in a 1-month pilot trial of FOCUS, a smartphone-based self-management intervention for SMI. At baseline and posttest, they completed measures examining symptoms and functional recovery. The participants provided qualitative feedback related to the usability and acceptability of the intervention.

**Results:**

Veterans completed on an average of 85.0 (SD 96.1) interactions with FOCUS over the 1-month intervention period. They reported high satisfaction, usability, and acceptability, with nearly all participants (16/17, 94%) reporting that they would recommend the intervention to a fellow veteran. Clinicians consistently reported finding mHealth-related updates useful for informing their care. Qualitative feedback indicated that veterans thought mHealth complemented their existing VA services well and described potential opportunities to adapt FOCUS to specific subpopulations (eg, combat veterans) as well as specific delivery modalities (eg, groups). In the 1-month period, the participants experienced small improvements in self-assessed recovery, auditory hallucinations, and quality of life.

**Conclusions:**

The FOCUS mHealth intervention is feasible, acceptable, and usable among veterans. Future work should develop and examine VA-specific implementation approaches of FOCUS for this population.

## Introduction

### Background

Serious mental illnesses (SMIs), including schizophrenia, bipolar disorder, and major depression, are associated with disruption of typical social [1] and vocational functioning [2], homelessness [3], and even premature death [4,5]. However, a significant portion of individuals with SMIs recover and enjoy long, productive, and meaningful lives [6]. A critical determinant of recovery is the capacity for symptom self-management, or coping with the illness to mitigate its negative effects. A growing body of evidence supports the effectiveness of self-management interventions for individuals with SMIs [7,8]. These interventions, which provide support and resources to facilitate coping skills and medication adherence, are associated with reductions in symptoms and risk of hospitalization, as well as increased recovery and quality of life [9]. The Department of Veterans Affairs (VA)—the nation’s largest integrated health care provider—has emerged as a leader in psychosocial rehabilitation for people with SMIs [10]. VA services, which include primary care, hospital medicine, and a comprehensive collection of specialty services, reach >9 million enrolled veterans each year [11]. SMI is overrepresented in VA health care settings relative to the general population [12], and veterans with SMIs are at increased risk of negative outcomes relative to other mental disorders [13]. Specifically, veterans with SMIs are at increased risk of comorbid chronic pain conditions [14], obesity [15], and undiagnosed and untreated trauma-related symptoms [16]. The constellation of medical and psychiatric complications associated with SMIs results in these individuals losing on average >14 years of life relative to the average [17].

Several barriers limit the reach and effectiveness of self-management interventions even among veterans receiving care in integrated health care systems such as the VA. First, veterans with SMIs face many challenges with care, including transportation, recall of appointment times, and the impact of personal crises on access to services [18]. Research suggests that very few individuals with SMIs receive specialized evidence-based psychosocial care for SMIs [19], and veterans living farther from VA health care facilities have poorer use [20]. Second, even when resources are available, veterans with SMIs are susceptible to disengagement. In total, 2 studies examining veterans with SMIs being treated at the VA found that, respectively, 42% and 47% of individuals with SMIs receiving care within the VA experienced a service gap of at least a year [20,21]. Third, even when individuals have access to and motivation to engage in care, typical in-person services are provided weekly or monthly. Self-management is most effective when it is activated immediately in response to stressors.

Recent developments in web-based and mobile technology have the potential to economically expand the reach and effectiveness of self-management interventions [22]. Individuals with SMIs report similar access to technologies as the general population [23] as well as an interest in the use of these technologies for mental health support [24]. A mobile health (mHealth) intervention for individuals with SMIs—FOCUS—has demonstrated usability among individuals with SMIs [25] and feasibility in community mental health settings [26]. A recent randomized trial comparing FOCUS with an evidence-based in-clinic group intervention for symptom self-management demonstrated comparable positive clinical effects between the 2 interventions, and those randomized into FOCUS remained engaged at higher rates than those randomized into typical in-person care [27].

The VA has also demonstrated innovations in the deployment of mHealth for mental health. A recent meta-analysis [28] identified 20 mental health or addiction mobile apps developed by the VA or the Department of Defense. Although these apps cover a variety of clinical interventions (eg, cognitive behavioral therapy [CBT] for insomnia; *CBTi Coach*), self-management activities (eg, tracking; *T2 Mood Tracker*), or diagnoses (eg, posttraumatic stress disorder [PTSD]; *PTSD Coach*), few (eg, *Virtual Hope Box* and *PTSD Coach*) have been tested in randomized trials and demonstrated significant improvements relative to waitlist [29] or usual care control conditions [30]. Many veterans report openness to using digital interventions for managing mental health [31], and over half of veterans receiving care for PTSD with access to digital technologies report interest in using mHealth for a range of clinical issues [32], although knowledge of available mHealth options remains a barrier to broad uptake among veterans.

There is a lack of mHealth tools designed for SMIs available through the VA. Of the apps currently featured on the VA mobile app website, none provide content specifically designed for the management of psychosis [33]. Although early work examining mHealth for SMIs has demonstrated its feasibility and effectiveness, there may exist specific features relevant to the deployment of these tools for veterans or within VA health care settings. Veterans with schizophrenia often present with comorbid chronic pain [14], other chronic medical conditions (eg, hypertension or diabetes) [34,35], or PTSD [36], which, when co-occurring with schizophrenia, increases the risk of suicide [37]. Veterans and active duty service members with mental illnesses also appear particularly susceptible to stigma associated with mental illness [38], which could affect their willingness to engage in clinical services at brick-and-mortar facilities. Insights gleaned from the deployment of technological innovations in community settings may not generalize to VA settings given specific institutional structures and clinical workflows [39]. Taken together, these risk factors suggest a need for research that examines the feasibility and acceptability of mHealth among veterans with SMIs receiving outpatient care from a VA facility.

### Objective

This study reports the results of a pilot feasibility study of FOCUS deployed in a VA outpatient clinic for individuals with SMIs (ie, a Psychosocial Rehabilitation and Recovery Center [PRRC]). This clinic provides access to ongoing group therapies, individual therapy and case management, medication management, and the option to access related VA services, including vocational support. The results aim to determine whether mHealth is (1) feasible to deploy in a VA setting and (2) acceptable to veterans with SMIs, as well as to explore the preliminary effectiveness of this intervention among veterans with SMIs and determine whether the participants’ qualitative feedback suggests changes that would make mHealth for SMIs more appropriate and effective for the VA setting or the veteran population.

## Methods

### Participants

The study was reviewed and approved by the VA Puget Sound Health Care System Institutional Review Board. The participants were 17 individuals receiving treatment from an outpatient psychosocial rehabilitation clinic in a VA hospital in the Pacific Northwest. Potential participants were eligible for the study if they (1) had a serious and chronic mental illness (eg, schizophrenia-spectrum or mood disorder) with (2) current or past psychotic symptoms and (3) received their services at the PRRC. They were excluded if they (1) were incapable of providing informed consent or (2) had hearing, vision, or motor impairments that made it impossible for them to use a smartphone. Clinicians first shared information about the study with prospective participants and assessed their potential interest. With veteran authorization, study clinicians provided these referrals to the research team, who then contacted participants by phone to schedule their first visit.

### FOCUS mHealth Intervention

FOCUS comprises 3 components: a mobile app, a clinician dashboard, and an mHealth support specialist. The FOCUS mobile app includes brief, preprogrammed self-management interventions that can be accessed by the user on demand. Participants can do this in two ways: (1) on demand completing a brief ecological momentary assessment (EMA) item that provides them with a tailored intervention (if they indicate distress) or (2) via the *toolbox*, which provides users with access to specific skill practices without tailoring assessment. Self-management interventions are also accessed via prompts that remind participants to use FOCUS (a device notification that reads *Would you like to check in with FOCUS?*). On the basis of their responses to the EMA items, FOCUS delivers tailored in-the-moment interventions. For example, if a participant responds to a prompted assessment by selecting the option that they are bothered by the thought that their voices *know everything*, the system provides an example of a mental exercise designed to challenge the validity of that belief. These notifications are automatically deployed 3 times per day. Intervention categories include voices (cognitive and behavioral strategies to cope with auditory hallucinations), mood (behavioral activation and other cognitive exercises), sleep (sleep hygiene psychoeducation and relaxation exercises), social functioning (cognitive exercises for persecutory ideation, anger management, and social skill training), and medication use (reminders, behavioral tailoring, and psychoeducation). For the duration of the study, the FOCUS system prompted within 3 time frames daily (9 AM-1 PM, 1 PM-5 PM, and 5 PM-9 PM; exact times within those ranges were determined randomly by the system each day).

All participant use of the system was logged on the web-based clinician dashboard, which was reviewed at least weekly by the mHealth support specialist, a member of the research team tasked with tracking and supporting participant use of FOCUS and providing relevant updates to the VA mental health treatment team [40]. On weekly calls with each participant, mHealth support specialists were tasked with (1) providing technical support in case of app issues and (2) encouraging the personalized use of FOCUS skills for participants’ specific concerns. These calls were designed to last between 5 and 15 minutes. In this study, the mHealth support specialist also attended weekly meetings with the psychosocial rehabilitation mental health treatment team, providing brief (ie, <1 minute) updates related to each veteran enrolled in the study including an overview of (1) their use of FOCUS, (2) their responses to FOCUS items (ie, indicating symptoms and functioning), and (3) skills and support provided during weekly mHealth calls. This ensured that the members of the clinical team were aware of progress and relevant clinical changes to inform ongoing standard treatment. The mHealth support specialist was also available as needed to the primary treatment team to answer questions about FOCUS functions and content.

### Procedure

At the baseline visit, the participants were provided with a detailed overview of the study, were given the opportunity to ask questions, and provided written informed consent after completing a brief competency questionnaire. After providing consent, the participants completed baseline study assessments (described below) and then received an orientation to FOCUS. The participants were given the opportunity to use their own personal device if they had one that was compatible with FOCUS (ie, an Android device) and were lent a study device if they did not. If necessary for those using a loaned study device, the orientation also included instructions on the use of the device, for example, operations such as turning the phone on or off, how to use the touchscreen, or how to place phone calls. FOCUS notifications (ie, the daily reminders) prompted the participants to complete assessments and receive interventions tailored to the goals individually set at baseline related to areas that they identified as being relevant to their recovery. At posttest visits, the participants returned the study device (if necessary) and again completed the same battery of assessments in addition to assessments related to the usability of FOCUS and a brief semistructured interview soliciting qualitative feedback. The participants were compensated with US $40 for each of the 2 study visits.

### Measures

The participants completed a modified version of the System Usability Scale (SUS) based on previous work examining the feasibility and acceptability of FOCUS [26] to assess acceptability and feasibility. In addition to the conventional 26 items, we included items that assessed whether FOCUS required adaptation for a veteran population (eg, *FOCUS is appropriate for use with veterans* or *FOCUS was well integrated into my usual care at the VA PRRC*). We administered brief questionnaires to members of the primary clinician team when a client on their caseload was involved in FOCUS to assess the feasibility and acceptability of weekly updates to the clinical team, asking (1) whether they found FOCUS updates useful and (2) whether those updates affected their clinical care. Following the study assessment battery, the participants also responded to open-ended questions requiring them to expand upon their experience with the intervention. We reported on responses to the following items: (1) *What did you like about the app?* and (2) *What did you not like about the app?* to assess intervention acceptability and usability. For items regarding fit and adaptation to veterans, we reported on (1) *Would you recommend the app to a fellow veteran? Why or why not?* and (2) *What are ways this app could be improved for use specifically with veterans?* This interview was conducted face-to-face at the VA medical center in a private setting by a trainee clinical psychologist or a research study coordinator. Responses to each item were recorded by hand by the study coordinator.

A total of six different clinical or functional outcomes were assessed: depressive symptoms, auditory verbal hallucinations, persecutory ideation, insomnia, quality of life, and overall recovery. Depressive symptoms were assessed using the Beck Depression Inventory–Second Edition [41], a 21-item assessment of ranging symptoms of depression that is summed for an overall score. Auditory verbal hallucinations were assessed using the Hamilton Program for Schizophrenia Voices Questionnaire [42], a 13-item self-report questionnaire that assesses the frequency and severity of one’s experience of auditory verbal hallucinations within the past week. The Green Paranoid Thoughts Scale [43], a 32-item questionnaire covering thoughts about intentional threats from others, provided an assessment of persecutory ideation. Sleep quality was assessed using the Insomnia Severity Index [44], a 7-item scale assessing the extent and severity of current insomnia as well as satisfaction with one’s current sleep routine. Quality of life was assessed using the Quality of Life Enjoyment and Satisfaction Questionnaire [45,46], an 18-item assessment of satisfaction in various areas of one’s life, including social connections, work, and leisure. Finally, recovery was assessed using the Illness Management and Recovery Scale [47], a 15-item assessment of self-management and recovery developed to be consistent with the theoretical guidelines underlying Illness Management and Recovery [48], an evidence-based treatment program focused on independent, self-directed recovery.

### Data Analytic Plan

We first examined descriptive statistics among all participants on the SUS to examine the acceptability, usability, and satisfaction among veterans using the intervention. We then examined the qualitative responses to the postintervention interview prompts. In total, 2 raters (BB and JLN) reviewed all interview responses and independently created proposed response categories that unified a particular idea to analyze the participants’ perspectives on the open-ended items related to the FOCUS app. Units were defined as the collection of all words in a statement that conveyed a single idea or attribute. All disagreements were reconciled through discussion between the coders.

We reported pre–post descriptive statistics and effect sizes to examine the preliminary effectiveness of FOCUS among veterans participating in psychosocial rehabilitation. Although not powered for statistical significance testing, we conducted a series of paired sample 2-tailed *t* tests to explore whether during the 1-month study period the participants experienced improvements in depressive symptoms, auditory verbal hallucinations, persecutory ideation, sleep quality, self-reported quality of life, and self-reported recovery.

## Results

### Demographics

Participant characteristics are reported in Table 1. Our sample was predominantly White (11/17, 65%), male (12/17, 71%), and never married (9/17, 53%); reported a high school diploma (8/17, 47%) or associate’s degree (6/17, 35%) as the highest educational level; and had experienced between 1 and 5 psychiatric hospitalizations (10/17, 59%). Although the inclusion criteria encompassed a mood or schizophrenia-spectrum disorder with current or past psychotic symptoms, multiple participants reported a comorbid diagnosis of PTSD (6/17, 35%). Other frequent diagnoses were schizophrenia (4/17, 24%), schizoaffective disorder (5/17, 29%), and major depressive disorder (6/17, 35%). The participants’ average age was 55.12 (SD 13.02) years.

**Table 1:** Demographic characteristics of the study participants (N=17).

Characteristic			Values
Age (years), mean (SD)			55.12 (13.02)
**Gender, n (%)**			
	Female	5 (29)	
	Male	12 (71)	
**Diagnosis, n (%)**			
	PTSD^a^	6 (35)	
	Major depressive disorder	6 (35)	
	Schizoaffective disorder	5 (29)	
	Schizophrenia	4 (24)	
	Unspecified schizophrenia-spectrum or psychotic disorder	2 (12)	
	Bipolar disorder	1 (6)	
**Race, n (%)**			
	American Indian or Alaskan Native	1 (6)	
	Asian	2 (12)	
	Black or African American	3 (18)	
	White	11 (65)	
**Ethnicity, n (%)**			
	Hispanic	2 (12)	
	Non-Hispanic	15 (88)	
**Highest degree, n (%)**			
	High school diploma or GED^b^	8 (47)	
	Associate’s degree	6 (35)	
	Bachelor’s degree	2 (12)	
	Other	1 (6)	
**Marital status, n (%)**			
	Never married	9 (53)	
	Married	2 (12)	
	Divorced	6 (35)	
**Smartphone ownership, n (%)**			
	Yes	12 (71)	
	No	5 (29)	
**Lifetime hospitalizations, n (%)**			
	0	3 (18)	
	1-5	10 (59)	
	6-10	2 (12)	
	11-15	0 (0)	
	≥16	2 (12)	

^a^PTSD: posttraumatic stress disorder.

^b^GED: General Educational Development.

### Feasibility

On average, the participants completed 85.0 (SD 96.1, median 48.0) EMA interactions with FOCUS and did so on an average of 19.29 (SD 9.27) of 30 access days (mean 64.3%, SD 30.9%). These interactions directly lead to a brief intervention when users indicate distress. In addition to these interactions, the participants used the FOCUS Toolbox (ie, direct access to skills) an average of 49.0 (SD 42.5, median 33.0) times (timestamps of the FOCUS Toolbox uses were not collected, so this figure does not standardize use across participants to the first 30 days of access). All but 1 participant (16/17, 94%) completed all 4 weekly check-ins with the mHealth support specialist by phone. The participant who did not (1/17, 6%) completed 2 of the 4 possible weekly calls. With regard to weekly check-ins with the clinical team, of the 48 times a questionnaire was administered to a clinician with 1 or more clients enrolled in the program, the clinician reported that they found the FOCUS update useful all 48 (100%) times and that these updates affected their clinical care (eg, orienting toward particular clinical concerns and providing additional follow-up) 24 (50%) times.

### Acceptability and Usability

The responses to all acceptability-related questions on the SUS are shown in Table 2. Overall, the participants described the intervention as highly acceptable. Nearly all participants reported that they would recommend FOCUS to a friend (16/17, 94%), and most reported that they felt satisfied with FOCUS (15/17, 88%) and would use FOCUS if they had access to it (14/17, 82%). With regard to their experience of its usability, veterans also provided overall positive feedback as nearly all veterans reported feeling comfortable (16/17, 94%) and confident (15/17, 88%) using FOCUS as well as thinking that it was easy to learn (16/17, 94%) and easy to use (16/17, 94%). Very few participants reported that they found FOCUS to be complicated (1/17, 6%) or that they needed to learn a lot (1/17, 6%) or receive technical support to use it (2/17, 12%). Most of the sample reported that they felt that FOCUS helped them manage their symptoms (12/17, 71%).

The participants provided qualitative insights in response to questions related to what they liked and did not like about FOCUS. A prominent positive theme of acceptability involved access to self-management tools. The participants reported that they liked that FOCUS was consistently available to them, that they were able to access helpful tools in the moment, and that they could provide updates about current functioning without having to wait for an upcoming appointment with an in-person provider:

I liked always having it on me. The only time I didn’t was at church or the store. I like having it on me, documenting my symptoms. [Usually] I have to tell [my clinician] what’s going on in a month. With this, it was immediate, I knew someone was reading.

Participant 4

It’s like a 24/7 therapist in my pocket.

Participant 11

Other positive participant responses reported an increased propensity to engage in reflection and self-management when they were using FOCUS, identifying either specific skills that they found helpful or describing a general sense that they were more aware of and equipped for coping with symptoms in the moment:

The app helped me more quickly identify that I was hearing voices and that I needed and could do something about it.

Participant 15

I didn’t feel like it was completely diffusing my symptoms, but it was like having a safety checklist that told me what I should do when I was struggling- even if I’ve already tried the skills.

Participant 16

Many participants reported that they appreciated the positive and supportive messaging provided by the intervention:

I like that it was supportive. It had positive messaging and positive feedback.

Participant 10

I like that it helped me get into a more positive frame of mind, even if I was reluctant about it, even if I felt reluctant to change.

Participant 14

When the participants reported on characteristics that they did not like about the app, fewer consistent themes emerged. The participants most commonly reported on specific design features that would have personalized FOCUS to more directly meet their needs, for example, the addition of a back button or changing particular check-in items:

Once you go into the main screen and select a new skill, you can’t back out. Made me feel like I was reporting something that I didn’t want to report. Also, make this app available for iPhone.

Participant 12

I would’ve changed my prompts to check in with my sleep, it would ask me “how did you sleep last night?” That’s all I would change.

Participant 11

Some participants reported feeling bothered by prompt notifications and how responding to these notifications either felt invasive or forced them to pay closer attention to their phones:

Although it was useful, I sometimes wouldn’t like when it would ask me to check in. Seemed like an all-day thing. Maybe should have had more information.

Participant 1

Having to reach for the phone. It got annoying to be prompted to go to the app.

Participant 9

Other participants reported disliking the degree of specificity of the intervention content, although some differed on whether the intervention content was too specific or too broad and general to be applied:

Sometimes the app felt “wishy washy” or “soft” almost too positive. I would have like to have more time with the app to play with it more.

Participant 14

Some of the wording. The way they worded sometimes not really getting to the point, but also specific, instead of being broad. That would be better [to be more broad].

Participant 5

**Table 2:** Participant usability and acceptability ratings (N=17).

Item		Disagree or strongly disagree, n (%)	Neutral, n (%)	Agree or strongly agree, n (%)
**Acceptability**				
	I would recommend FOCUS to a friend.	1 (6)	0 (0)	16 (94)
	I found the check-ins with the mHealth^a^ specialist to be helpful.	0 (0)	1 (6)	16 (94)
	I am satisfied with FOCUS.	1 (6)	1 (6)	15 (88)
	If I have access to FOCUS, I will use it.	2 (12)	1 (6)	14 (82)
	I think that I would like to use FOCUS often.	2 (12)	2 (12)	13 (77)
	FOCUS is fun to use.	1 (6)	5 (29)	11 (65)
	I feel I need to have FOCUS.	3 (18)	4 (24)	10 (59)
**Usability**				
	The information provided for FOCUS was easy to understand.	0 (0)	0 (0)	17 (100)
	The mHealth specialist provided useful feedback on my use of the program.	0 (0)	0 (0)	17 (100)
	I felt comfortable using FOCUS.	0 (0)	1 (6)	16 (94)
	It was easy to learn to use FOCUS.	0 (0)	1 (6)	16 (94)
	How things appeared on the screen was clear.	0 (0)	1 (6)	16 (94)
	I thought FOCUS was easy to use.	0 (0)	1 (6)	16 (94)
	I felt very confident using FOCUS.	0 (0)	2 (12)	15 (88)
	Overall, I am satisfied with how easy it is to use FOCUS.	0 (0)	2 (12)	15 (88)
	I found that the different parts of FOCUS work well together.	1 (6)	2 (12)	14 (82)
	I was able to complete the modules quickly in FOCUS.	0 (0)	3 (18)	14 (82)
	It was easy to find the information I needed.	0 (0)	3 (18)	14 (82)
	Whenever I made a mistake using FOCUS, I could recover easily and quickly.	4 (24)	4 (24)	9 (53)
	I think that I would need the support of a technical person to be able to use FOCUS.^b^	12 (71)	3 (18)	2 (12)
	I found FOCUS to be very complicated.^b^	12 (71)	4 (24)	1 (6)
	I needed to learn a lot of things before I could get going with FOCUS.^b^	11 (65)	5 (29)	1 (6)
	I thought that there was too much inconsistency in FOCUS.^b^	15 (88)	2 (12)	0 (0)
	I found FOCUS very awkward to use.^b^	15 (88)	2 (12)	0 (0)
**Veteran fit and adaptation**				
	FOCUS is appropriate for use with veterans.	0 (0)	1 (6)	16 (94)
	I would imagine that most people would learn to use FOCUS very quickly.	1 (6)	2 (12)	14 (82)
	FOCUS was interactive enough.	5 (29)	4 (24)	12 (71)
	FOCUS helped me manage my symptoms.	3 (18)	2 (12)	12 (71)
	FOCUS was well integrated into my usual care at the VA^c^ PRRC.^d^	0 (0)	5 (29)	12 (71)
	FOCUS works the way I want it to work.	2 (12)	8 (47)	7 (41)

^a^mHealth: mobile health.

^b^Reverse-coded such that disagreement denotes higher perceived usability or acceptability.

^c^VA: Department of Veterans Affairs.

^d^PRRC: Psychosocial Rehabilitation and Recovery Center.

### Adaptation for Veterans

In addition to reporting that FOCUS was highly usable and acceptable, the participants provided information related to the fit of FOCUS for veterans and a VA outpatient mental health setting. Nearly all participants (16/17, 94%) reported that they felt FOCUS was appropriate for use with veterans, and most (12/17, 71%) reported that they felt FOCUS was well-integrated into their usual care at the VA.

The participants also provided additional information about the VA-specific application of FOCUS. At the start of the qualitative questions, the participants were asked whether they would recommend FOCUS to a fellow veteran, and their responses were almost uniformly affirmative (16/17, 94%). When asked to identify how FOCUS could be adapted to improve its acceptability among veterans, most participants reported that they had no suggestions for adaptations and that FOCUS nicely paralleled their current treatment needs as the intervention provides access to similar skills to those emphasized in VA outpatient services:

There are vets that have seen combat, war, and this FOCUS app would be a good resource to help curb the PTSD they might develop. Helped me be more positive and helped me realize my moods, and helped remind me to take my meds. This will help open people’s minds to being more open to getting help...It was a good experience and it’s good for veterans and it’s a positive influence tool to help the veteran in their therapy.

Participant 12

Veterans can help find a way to subside the voices, because the app will help them. They just have to listen to the app’s suggestions.

Participant 15

The participants specifically emphasized that FOCUS was helpful in reducing negative thinking and decreasing stress and that these characteristics were particularly well-suited to a veteran population:

I think it would help people. If you have a lot of negative thoughts you can check in with yourself and get out of your head.

Participant 13

With regard to improvements and adaptations for veteran populations, the participants commonly identified adaptations that would improve FOCUS for subpopulations of veterans, for example, veterans with hearing impairments or PTSD:

A way for hearing and vision impaired veterans to be able to use the app. I can’t think of how but a way for them to use the app too.

Participant 14

Have more solutions, more things going on. More content. Maybe for PTSD. These guys have a hard time, probably worse than I have. PTSD support.

Participant 4

Expand the voices option. I think people with PTSD hear things in their own head. That would be an improvement.

Participant 5

A second emergent theme involved integrating FOCUS more closely into existing VA services. Notably, on the SUS, fewer participants reported that they felt FOCUS was well-integrated into their routine services than those who reported that they enjoyed the use of the app or mHealth coaching. Some participants commented on connecting FOCUS to existing structures, including referral services or group meetings:

Connecting it to existing care, like having an mHealth referral service in VA. The doctor could recommend it to a veteran, and then a coordinator picks it up.

Participant 2

Hold group meetings for FOCUS, to get together with other veterans to discuss and share how everyone is managing their symptoms. We could compare notes with each other. We need more apps like this for veterans.

Participant 15

### Clinical Outcomes

The summary statistics of the models examining clinical outcomes are reported in Table 3. Paired sample *t* tests were conducted to examine within-participant changes during the 1-month study period. Given that the primary aim of this pilot study was to establish the feasibility and acceptability of this approach in a VA setting, these analyses were underpowered to detect significant clinical effects; however, we report the effect sizes here. Small positive effects were detected for participants in self-directed recovery (Illness Management and Recovery Scale; Cohen *d*=0.30), quality of life (Quality of Life Enjoyment and Satisfaction Questionnaire; Cohen *d*=0.25), and severity of the voices (Hamilton Program for Schizophrenia Voices Questionnaire; Cohen *d*=0.23).

**Table 3:** Baseline and posttest scores of clinical outcome measures.^a^

Clinical outcome measure	Baseline score, mean (SD)	Posttest score, mean (SD)	Difference, mean (SD)	*t* test (*df*)	*P* value	Cohen *d*
Recovery (IMRS^b^)	34.71 (5.65)	35.94 (6.67)	−1.24 (4.19)	1.22 (16)	.24	0.30
Quality of life (QLES-Q^c^)^d^	49.44 (9.02)	51.31 (6.73)	1.88 (7.64)	0.98 (15)	.34	0.25
Voices (HPSVQ^e^)	20.50 (5.68)	19.20 (6.32)	1.30 (5.35)	0.77 (9)	.46	0.24
Insomnia (ISI^f^)	11.35 (6.12)	10.71 (5.75)	0.64 (5.06)	0.53 (16)	.61	0.13
Depression (BDI-II^g^)^d^	25.44 (13.93)	24.50 (9.37)	−0.94 (7.69)	0.49 (15)	.63	0.12
Medication beliefs (BMQ^h^)	11.00 (11.18)	11.53 (10.52)	0.53 (5.36)	0.41 (16)	.69	0.10
Paranoia (GPTS^i^)^d^	67.63 (30.71)	69.94 (32.77)	2.31 (22.72)	0.41 (15)	.69	−0.10

^a^All the effects were statistically nonsignificant. Effect sizes are computed such that positive values reflect changes in the expected direction.

^b^IMRS: Illness Management and Recovery Scale.

^c^QLES-Q: Quality of Life Enjoyment and Satisfaction Questionnaire.

^d^Because of missing data from skipped items, N=16 for analyses involving the Beck Depression Inventory–Second Edition, QLES, and Green Paranoid Thoughts Scale.

^e^HPSVQ: Hamilton Program for Schizophrenia Voices Questionnaire. HPSVQ scores reported are those of participants (n=10) who reported any level of auditory verbal hallucinations at baseline and completed the study.

^f^ISI: Insomnia Severity Index.

^g^BDI-II: Beck Depression Inventory–Second Edition.

^h^BMQ: Brief Medication Questionnaire.

^i^GPTS: Green Paranoid Thoughts Scale.

## Discussion

### Principal Findings

This study aimed to examine the feasibility, acceptability, usability, and preliminary effectiveness of the FOCUS mHealth intervention in a VA psychosocial rehabilitation outpatient setting. The participants used FOCUS frequently during the month-long deployment period (mean 85.0, SD 96.1 assessed interactions and mean 64.3%, SD 30.9% of days enrolled in the study) and overwhelmingly reported that they found the intervention acceptable and usable. This matches previous work examining the acceptability of FOCUS in non-VA populations [49]. When asked to elaborate on adaptation for the VA setting, veterans largely found the intervention ready to deploy, but a few participants provided suggestions for improvement, including content for specific veteran subpopulations (ie, PTSD or sensory impairments) as well as integration into existing services (ie, referral services and mental health groups). The trial was underpowered to detect statistically significant changes in clinical outcomes, and the effect sizes were consistent with small improvements. Together with existing research supporting the effectiveness of a 3-month deployment of FOCUS [27], this pilot study suggests that the FOCUS mHealth intervention is appropriate for a large-scale trial in a VA setting to evaluate effectiveness.

Use statistics suggested that the participants were able to access a substantial weekly dose of the FOCUS clinical intervention during the 1-month study period. The participants also almost unanimously completed a weekly FOCUS check-in call every week that they were enrolled. This high rate of use mirrors previous studies of FOCUS, including among those with a recent psychiatric hospitalization and individuals enrolled in outpatient community mental health [50]. These use rates are particularly notable in a veteran population given the low rates of veteran use of existing VA or Department of Defense mental health apps [31]. These results suggest that a usability-tested mHealth intervention such as FOCUS, together with weekly mHealth support and coaching from a member of the study team, could sufficiently engage veterans enrolled in outpatient mental health services.

Regarding fit for veterans, many participants reported feeling that FOCUS symptom management skills closely mirrored their current mental health treatment, particularly in its impact on reducing unhelpful negative thinking. Some participants provided recommendations for VA-specific adaptations, including subpopulation-relevant content (eg, comorbid PTSD support) and creating referral pathways for mHealth provision, as well as the development of mental health groups where veterans can practice FOCUS skills in a socially supportive format. Despite a growing body of evidence, few mHealth interventions have been implemented in real-world practice. As one of the nation’s largest health care providers, the VA could provide fertile ground for testing of various mHealth implementation models, including, for example, an embedded mHealth specialist in primary care or a supportive group in outpatient mental health. Future hybrid and implementation-oriented work could identify the specific organization-related variables linked with the most successful VA deployments of mHealth for SMIs.

The participants’ overall ratings of the usability and acceptability of the intervention were high and closely mirrored comments regarding acceptability in non-VA community mental health settings [49]. All but 1 participant (16/17, 94%) reported that they would recommend FOCUS to a friend and that FOCUS was appropriate for use with veterans. Qualitative responses suggested that the participants particularly appreciated the positive tone of the messages, the symptom management skills delivered, its around-the-clock availability for support, and its simplicity and straightforward design. In addition to these positive comments, the participants reported on features of the intervention that they did not enjoy, including specific design features (eg, the inability to go *back* and having limited time to respond to prompts) and being interrupted by device notifications from FOCUS, as well as suboptimal degree of specific versus broad app content (though this varied across participants as to which was preferred). On one hand, these specific points of feedback were relatively uncommon, and most participants reported high levels of satisfaction with the FOCUS app itself. In contrast, FOCUS could benefit from improved personalization and fit to the user’s specific needs and preferences. Future innovations could allow for automated customization to meet this objective.

The clinical effects were smaller than those reported in other clinical trials examining FOCUS [26]. At posttest, the participants experienced small but nonsignificant improvements in recovery, quality of life, and severity of auditory hallucinations. The study sample may have affected these results. The participants enrolled in this trial were well-established in a psychosocial rehabilitation program, and FOCUS was provided as an adjunct to existing services. The participants were not required to be naive to the interventions on which FOCUS was based (eg, CBT for insomnia, CBT for psychosis, and social skill training), and many reported that the intervention content mirrored care they had already received. Furthermore, the participants received 1 month of the FOCUS intervention rather than the 3-month period that has been suggested as standard in full-scale trials [27]. It is possible that treatment effects would have been larger after a full course of the intervention.

Other study limitations warrant mention. Given the small sample size and brief study period, our findings speak primarily to the feasibility, acceptability, and usability of FOCUS in a veteran population. Conclusions related to clinical benefits cannot be drawn. Second, the clinical model for this deployment of FOCUS involved weekly calls from a member of the study team. This model may have limited generalizability to clinics where frontline clinicians may be operating in this mHealth clinical support role. Furthermore, although updates were provided to the participants’ mental health clinicians, there was no specific protocol in place to make FOCUS data actionable. Given the brief length of the trial, many participants also reported that they did not meet with their primary clinician for an individual session during the intervention period; therefore, the benefits of ongoing FOCUS assessments to routine care were not explicitly examined. Finally, in general, given the multiple components of the intervention (ie, mobile app, weekly check-in calls, and communication with the primary clinical team), it will be difficult to know without more rigorous trials the extent to which any clinical gains might be attributable to particular components of the intervention. Future work should also examine whether benefits might differ in various subgroups of veterans, including those with varying degrees of digital literacy.

### Conclusions

Overall, the results suggest that FOCUS is feasible, acceptable, and usable to a veteran population. Future randomized effectiveness and hybrid trials can provide insight into the specific adaptations to ensure successful implementation of mHealth for SMIs in the VA population. If effective, FOCUS could fill a critical gap in the currently available suite of VA mobile apps and has potential for significant impact on the VA. This study suggests that future work is warranted and provides initial suggestions for such efforts.

## Abbreviations

CBT: cognitive behavioral therapy
EMA: ecological momentary assessment
mHealth: mobile health
PRRC: Psychosocial Rehabilitation and Recovery Center
PTSD: posttraumatic stress disorder
SMI: serious mental illness
SUS: System Usability Scale
VA: Department of Veterans Affairs
